# Findings of Breast Sonography in Patients with Foal Asymmetric Breast Density on Mammography

**Published:** 2011-06-01

**Authors:** Z Zare, T Faghihi Langroudi

**Affiliations:** 1 Department of Radiology, Shiraz University of Medical Sciences, Shiraz, Iran

**Keywords:** Asymmetric, Breast, Density, Mammography, Sonography

## Abstract

**Background:**

The imaging parameters that mandate further diagnostic workup in focal asymmetric breast densities are not clearly defined. To identify indications for further workup in Focal asymmetric breast densities (FABD) by doing ultrasonography.

**Methods:**

One-hundred women underwent breast ultrasonography after incidental discovery of FABD on mammograms. Mammograms and sonograms were evaluated for lesion location, associated calcifications, architectural distortion and change from previous examination when available.

**Results:**

Twenty three patients had abnormal sonographic findings and the site of sonographic abnormal findings was the same as the site of FABD on mammography. Sonographic findings were 7 focal increases in fibrous tissue, 5 ductal ectasias,4 simple cysts, complex cyst in one, 4 benign solid masses, one malignant solid mass and one with fibrous tissue at the site of pervious breast surgery. There was a significant relation between FABD in upper inner quadrant and normal sonography (p=0.036) and FABD in retroareolar region and ductal ectasia in sonography (p=0.002).

**Conclusion:**

FABD usually present a benign etiology and can safely be managed by follow up. Sonography helps the physician do tissue diagnosis by detecting mass with features of possibly malignancy, in the women with negative physical examination.

## Introduction

Focal asymmetric breast densities (FABD) are defined relative to the contrateral breast.

The American College of Radiology Breast Imaging Reporting and Data System (BI-RADS) lexicon includes it as "Asymmetry of tissue density with similar shape on two views but completely lacking borders and the conspicuity of a true mass".[1] It is found in approximately 3% of mammograms.[2] In the past, asymmetric breast tissue was regarded as a mammographic sign of malignancy.[3] A review of the literature revealed that the rate of malignancy of asymmetric breast tissue found the biopsies is 0-14%.[4][5][6][7][8][9] Because some masses with either ill-defined borders or obscured borders by surrounding fibroglandular tissue may represent as focal asymmetric densities, so further imaging evaluation of focal asymmetric density detected on mammography, may be essential. Data on ultrasonographic findings in breasts with

FABD are scarce and the results are conflicting. One study of eight patients showed that most of their sonograms were normal,[2] while another study of 15 patients found a sonographic abnormality in 6 cases.[10] To the best of our knowledge, there are no published large series correlating mammographically detected FABD with sonographic findings. The purpose of this study was to review the spectrum of clinical, mammographic and sonographic findings of FABD.

## Materials and Methods

Between January 2007 and January 2008, 100 women who presented for either screening or diagnostic mammography were identified as having benignappearing asymmetric breast tissue (as defined by the American College of Radiology's BI-RADS lexicon). Clinical data included the patient's age, use of hormone replacement therapy, close family history of breast cancer, history of birth control pills taking, parity, and the presence of a any palpable mass.

Mammograms and sonograms were evaluated for lesion location, associated calcification, architectural distortion and axillary lymph nodes. The patients who were being treated with hormonal replacement therapy or those with palpable masses had not been considered for this study. No patient had a history of biopsy or any other form of major trauma to the affected breast. The mammograms were obtained with a GE sonographe 600T senix HF mammography unit. All patients underwent routing mammography, which consisted of craniocaudal and mediolateral oblique views. The US examinations were performed by using logic 7 (GE: medical systems) with a 7.5-MHZ linear–array transducer. Statistical Analysis was performed using SPSS software (Version 15, Chicago, IL, USA). Data of mammographic and sonographic records were compared using the t test. Other tests such as Mann Whitney test, Fisher's exact test and Chi-Square were also used.

## Results

All patients, based on the location of asymmetric tissue were classified into 6 groups including Central, UOQ, UIQ, LOQ, LIQ and Retroareolar ones. In 77 cases (77%), no abnormality was identified and only normal appearing tissue was visualized. In 7 cases (30.43% of abnormal US findings), the tissue in question appeared heterogeneous but predominantly echogenic relative to the surrounding fatty tissue and regarded as prominent fibrous tissue. In 5 cases, (5% of the entire patient, 21.4% of abnormal US findings), there was linear hypoechoic structures that were suggestive of ductal ectasia (5% of the entire patient, 21.27% of abnormally sonographic findings). Five solid masses were detected, 4 of these fines (17.39% of abnormal sonographic findings) had a benign appearance, and another one (4.35% of positive sonographic findings) had features of a possible malignancy, hypoechoic with an ill-defined border without acoustic shadowing, and it was proved on pathology to be malignant.

Five cystic masses were also found, 4 of them had benign looking appearance and only one of them had a complex feature. The patient refused to undergo biopsy and subsequently had been followed up. One of the patients (4.35% of US abnormal findings) had only fibrous tissue at the site of previous benign mass operation. All patients with FABD in upper inner quadrant had normal sonographic findings. There was a relevant relation between them (p=0.036). Eighty percent of patients with retroareolar asymmericity had ductal ectasia on sonogram. Using Fisher Exact test, there was a correlation between them (p=0.002).

## Discussion

Previous reports have described a wide spectrum of both benign and malignant entities that may be responsible for FABD. Deciding which lesions need further evaluation is often clinically challenging. It was suggested that FABD that does not form a mass or are not associated with architectural distortion, clusters of calcification or a clinically palpable mass are most probably a benign variation of the norm and could be safely followed.[11]

In our study, one of the patients had a malignant mass. Actually, US should be used to rule out the diagnoses of other radiographic findings (e.g. whether a mass is benign or malignant). As Sperber et al. stated, when sonography could not define a lesions, the pathology was benign and when sonography diagnosed a solid mass, the pathology carried a higher probably of malignancy.[2] In the one study of Piccoli et al. on the 8 patients with FABD, all had normal sonographic findings, although it should be mentioned that the numbers of their sample was too low in comparison to us to detect any abnormality.[2] Rissaren et al. reported on 15 patients that 47% had abnormal sonographic findings. However, this study, too, is of limited value because of low number of cases.[10] Finally Shetty et al. described a retrospective series of 36 patients, 73% of which had abnormal sonographic findings.

Their study was limited principally by its retrospective design, and the second one was patient's selection since all of their patients underwent biopsy. However FABD purse is not an indication for biopsy without the other findings such as palpable mass and architectural distortion. This study overcomes the limitations of prior investigations because it is more completely prospective study. Six of our patients had previous mammograms that were available for comparison. Two of them had a new or growing FABD, but with normal sonographic findings.

Previous studies however had shown that new or growing FABD should be managed with caution. Due to the small number of malignant lesions and lack of previous mammograms, we cannot assess the overall probability of malignancy growing or new FABD form our data. In conclusion, in our study most of the FABD had no abnormal findings on sonogram, but if sonography detects suspicious solid masses, it mandates further diagnostic evaluation such as biopsy, so assessment with sonography is useful to permit detection of a non-palpable solid mass earlier and then further investigation must be performed. Further studies on the radiologic-pathologic correlation and follow up mammographic of FABD are warranted to achieve more precious in defining radiological findings.
